# Does decreasing serum uric acid level prevent hypertension? – a nested RCT in cohort study: rationale, methods, and baseline characteristics of study cohort

**DOI:** 10.1186/1471-2458-13-1069

**Published:** 2013-11-12

**Authors:** Kun Song, Yuan Wang, Guolin Wang, Qing Zhang, Huanli Jiao, Guowei Huang, Wenli Lu

**Affiliations:** 1 Health Management Center, General Hospital, Tianjin Medical University, Tianjin, China; 2 Department of Health Statistics, School of Public Health, Tianjin Medical University, Tianjin, China; 3 Department of Nutrition and Food Hygiene, School of Public Health, Tianjin Medical University, Tianjin, China

**Keywords:** Randomised controlled trial, Cohort study, Pre-hypertension, Hyperuricemia

## Abstract

**Background:**

Previous epidemiologic studies have demonstrated an association between uric acid and hypertension. Our objective was to conduct a prospective cohort study with a nested randomised controlled trial (RCT in cohort) that aims to identify the association of hyperuricemia with the development of hypertension and to examine the efficacy of dietary intervention in lowering uric acid level and prevention of hypertension.

**Methods/Design:**

Participants were considered eligible to enrol for this cohort study if they were not diagnosed with hypertension until their last routine health examination. The characteristics of the eligible participants were analyzed. After enrolment, participants with prehypertension and hyperuricemia simultaneously were randomly distributed to either the intervention group or the control group. An education package of dietary intervention for lowering uric acid was delivered to the intervention group. The primary evaluation criterion was the first manifestation of hypertension.

**Discussion:**

Based on the results of their health examination in 2010, 19, 724 subjects met the inclusion criteria and this source population guaranteed the required minimum sample size for this study. The baseline characteristics of the study cohort showed that hyperuricemia was associated with prehypertension, and was independent of age, body mass index (BMI), and abdominal obesity in females; however, in males it was contrary.

The impact of lowering uric acid on the prevention of hypertension is still inconclusive. This RCT in cohort study provides important data on the prevention of hypertension, especially in patients with a high risk for hypertension development. Results are expected to be available in 2015.

**Trial registration:**

The study is registered with the Chinese Clinical Trial Registry (ChiCTR-TRC-12002925).

## Background

Previous epidemiologic studies have demonstrated an association between uric acid and hypertension [1]. Experimental evidence supports a causal role of serum uric acid in hypertension development [2]. Higher serum uric acid levels were also positively associated with prehypertension when hypertension prevention efforts may be applicable independent of smoking, body mass index (BMI), diabetes, and other confounders [3]. In addition, a significant decrease in the systolic blood pressure was observed in patients with hyperuricemia and hypertension after a reduction in the intake of food sources containing purines [4]. Based on the existing body of evidence, it appears that hyperuricemia patients with high normal blood pressure would benefit from lowering uric acid dietary intervention for the prevention or postponement of the development of hypertension. There seems to be sufficient evidence to warrant further studies to determine whether lowering uric acid levels would be of clinical benefit in the prevention of hypertension [5-10]. In this article, we describe a prospective cohort study with a nested randomised controlled trial (RCT in cohort) that aims to examine the association between uric acid level and hypertension occurrence.

## Methods/Design

### Study design

The study is a prospective cohort study with a nested randomised controlled trial. The objectives are to identify the association of hyperuricemia with the development of hypertension and to examine the efficacy of dietary intervention in lowering uric acid level and its impact on prevention of hypertension. Participants were randomly enrolled in this study cohort when they had their yearly routine health examinations in the department of Health Examination at the General Hospital in Tianjin, China in 2010. Participants who had hypertension were excluded. 19, 724 subjects met the inclusion criteria. The study cohorts include the following four groups: normal tension and no hyperuricemia (NN), prehypertension and no hyperuricemia (PN), normal tension and hyperuricemia (NH), NH)and prehypertension and hyperuricemia (PH). After enrolment, participants who had prehypertension and hyperuricemia were randomised either to the intervention group (PHI) or the control group (PHC). Participants were randomized by a computer-generated number, which was concealed in sequentially numbered, sealed, and opaque envelopes, and kept by a trained nutritionist who delivered the intervention. Ethical approval was obtained from the ethical committee of the Tianjin Medical University (Additional file 1). Signed informed consent was obtained from all the participants (Additional file 2).

### Investigational plan

Table 1 summarizes the investigation plan. The cohort will be followed for at least for 5 years. Baseline demographic information including age, gender, social history and habits, and family history was collected after enrolment. Subjects were asked to have their annually scheduled health examination and finish a questionnaire on lifestyle factors and medical history at each scheduled health examination. A question was designed to investigate average salt intake of family members and a supplemental question was used to identify if the participant favored more or less salty food than other family members. The patients were asked if they were diagnosed with hypertension in the previous period before the scheduled health examination and the time was documented. Subjects in RCT for both the intervention and control groups were asked to attend three additional scheduled visits at 3 months, 6 months, and 18 months after the first study health examination.

**Table 1:** Documentation and investigation plan

**Documentation or investigation**	**Baseline**	**3 Mo**	**6 Mo**	**1 Yr**	**18 Mo**	**2 Yr**	**3 Yr**	**4 Yr**	**5 Yr**
Consent form	W								
Questionnaire-demographic information	W								
Questionnaire-lifestyle factors and medical history	W	R	R	W	R	W	W	W	W
Blood pressure	W	R	R	W	R	W	W	W	W
Blood screening-Uric acid	W	R	R	W	R	W	W	W	W
Laboratory values: Total cholesterol, LDL-C, HDL-C, TG, Blood glucose	W			W		W	W	W	W

LDL-C: Low-density lipoprotein concentrations, HDL-C: High-density lipoprotein concentrations, TG: Triglycerides.

### Description of intervention in RCT

Participants were asked to answer questionnaires about their dietary habits and lifestyle. After evaluating the participants’ dietary habits and lifestyle, a trained nutritionist will make tailored recommendations for each participant. An information letter that included the tailored recommendations was sent to the subjects after the face-to-face health-related advisory service. Generally, three modules were included: risk of hyperuricemia, dietary recommendations with lists of restricted and recommended food according to the subject’s recollection, and lifestyle modification recommendations.

### Measurements and definitions

A trained nurse measured blood pressure under controlled conditions by using a portable digital blood pressure monitor (Omron HEM-705CP). Blood pressure was measured three times in succession with the subject seated, with the left arm at heart level, and cuff adjusted for arm circumference. For each subject, the mean of the second and third readings was used for the analysis of systolic blood pressure (SBP) and diastolic blood pressure (DBP) data. Prehypertension is defined as the SBP between 120 and 139 or DBP between 80 and 89 mm Hg without using anti-hypertension medication according to the Seventh Report of the Joint national Committee on Prevention, Detection, Evaluation, and Treatment of High Blood Pressure guidelines. Hypertension is defined as SBP > 140 or DBP > 90 mm Hg or using anti-hypertensive medication.

Body mass index (BMI) is used as an index of relative weight. Waist circumference (WC) is used as a measure of central obesity. The data on height, weight, and waist circumference are based on the mean of two measurements. Overweight is defined as BMI ≥ 24 kg/m^2^, and obesity as BMI of ≥ 28 kg/m^2^ while abdominal obesity is defined as WC > 90 cm in male and 85 cm in female. Venous blood samples were obtained after an overnight fast and they were subjected to centrifugation at 3000 rpm for 30 minutes at 4°C. Serum uric acid, triglycerides (TG), high-density lipoprotein (HDL) and low-density lipoprotein (LDL) concentrations, and fasting glucose were determined enzymatically by an auto-analyzer (Hitachi Model 7170 analyzer, Hitachi, Tokyo, Japan). Hyperuricemia is defined as a serum uric acid value above 6.0 mg/dL for females or above 7.0 mg/dL for males. Renal function was assessed by testing creatine, creatinine-urine, creatine clearance, and BUN.

### Estimation of minimum sample size

For the consideration of the RCT design, sample size calculation for the primary endpoint was made under the assumption that during the 5-year observational period about 30% of the subjects in the intervention group and 20% of the subjects in the control group will develop hypertension. The detectable risk increase is compatible with 80% power and the significance of 0.05 is 10%. Based on this assumption, a sample size of n = 289 was required for each group. For the NN, PN, and HN groups, we adopted a minimum sample size, which is the same as that for each RCT group. The targeted sample size for the enrolment was estimated with the minimum sample size for the five groups (n = 1445) multiplied by 1.2 with the consideration for the loss of participants during the follow–up period. In total, the targeted sample size was 1800.

### Outcome and statistical analysis

The primary evaluation criterion for the present study is the manifestation of hypertension. Cox proportional-hazards regression was used to identify the association between baseline uric acid levels with the occurrence of hypertension in the non-intervention cohort. We used mixed models since we had correlated repeat observations of uric acid, blood pressure, and lipid profile. We then, compared the change in BMI, uric acid, and blood pressure from the baseline to five years for each subgroup.

### Baseline characteristics of the eligible subjects

Based on the results of their health examination in 2010, 19, 724 subjects met the inclusion criteria including 10, 423 males and 9, 301 females. The average age of the participants was 40.1 ± 13.1 years (range, 14 to 92 years). Baseline characteristics are presented in Table 2. The required minimum sample size was guaranteed. Individuals aged from 35–75 years were asked if he or she would participate in their scheduled annual health check-ups.

**Table 2:** Baseline characteristics of eligible study participants

**Variables**	**NN**	**PN**	**NH**	**PH**
n	8542	8687	917	1578
Age ($\bar{x}\pm s$, yrs)	37.1 ± 11.4	42.7 ± 13.9	38.3 ± 12.0	42.7 ± 14.1
Gender (Male %)	39.8	60.6	63.1	75.1
BMI ($\bar{x}\pm s$, kg/m^2^)	22.8 ± 3.1	24.8 ± 3.4	24.5 ± 3 .3	26.5 ± 3.4
Waist circumstance ($\bar{x}\pm s$, cm)	75.2 ± 13.2	82.2 ± 10.4	81.5 ± 10.4	87.5 ± 9.9
Sbp ($\bar{x}\pm s$, mm Hg)	106.7 ± 6.9	124.3 ± 6.4	108.2 ± 6.4	124.7 ± 6.6
Dbp ($\bar{x}\pm s$, mm Hg)	67.7 ± 5.7	78.4 ± 5.7	68.9 ± 5.4	79.2 ± 5.5
Serum uric acid ($\bar{x}\pm s$, mg/dl)	4.7 ± 1.0	5.0 ± 1.1	7.3 ± 0.8	7.5 ± 0.9
Total cholesterol ($\bar{x}\pm s$, mg/dL)	4.9 ± 0.9	5.2 ± 1.0	5.3 ± 1.0	5.4 ± 1.0
Triglycerides ($\bar{x}\pm s$, mg/dL)	1.1 ± 0.9	1.5 ± 1.3	1.9 ± 1.4	2.2 ± 1.7
LDL ($\bar{x}\pm s$, mg/dL)	3.1 ± 0.8	3.3 ± 0.9	3.4 ± 0.9	3.3 ± 0.9
HDL ($\bar{x}\pm s$, mg/dL)	1.4 ± 0.3	1.4 ± 0.3	1.3 ± 0.3	1.3 ± 0.3
Fasting glucose ($\bar{x}\pm s$, mg/dL)	4.7 ± 0.8	4.9 ± 1.1	4.9 ± 1.0	5.0 ± 0.9
Diabetes (%)	1.8	4.1	3.2	4.0

*Normaltension and no-hyperuricemia (NN), Prehypertension and no-hyperuricemia (PN), normaltension and hyperuricemia (NH)and Prehypertension and hyperuricemia (PH).

The prevalence of prehypertension and hyperuricemia was 52.0% (n = 10,265) and 12.6% (n = 2495), respectively. The prevalence of prehypertension was higher for male (61.9%) than that for female (41.0%, chi-square = 854.0, p < 0.001). The prevalence of hyperuricemia was higher for male (16.9%) than that for female as well (7.9%, chi-square = 365.5, p < 0.001). Overweight and obese subjects had a high prevalence of prehypertension (65.0% and 78.6% in male; 55.5% and 70.6% in female). Subjects with abdominal obesity were more likely to have high-normal blood pressure than subjects without abdominal obesity (56.5% vs. 75.4% in male and 37.8% vs. 69.2% in female). Subjects with hyperuricemia had a higher prevalence than subjects without hyperuricemia (60.8% and 67.2% in male; 40.0% and 53.8% in female) (Table 3). The association of prehypertension with age was stronger for female than male as the odds ratio was 1.01 (95% CI: 0.91–1.09), 2.71 (95% CI: 2.12–3.48) for middle and old age male groups and 1.93 (95% CI: 1.76–2.12), and 7.29 (95% CI: 5.40–9.83) for middle and old age female groups. Hyperuricemia was significantly associated with prehypertension independent of the age group, BMI, and abdominal obesity among female subjects in a forward multiple logistic regression analysis, however this was not the case among the male subjects (Table 4).

**Table 3:** The prevalence of prehypertension in subgroups regarding to Age, BMI, Abdominal Circumference and Hyperuricemia

**Variables**	**Male**					**Female**				
	**N**	**n**	**%**	***χ***^**2**^	***P***	**N**	**n**	**%**	***χ***^**2**^	***P***
Age group										
Young (18–34)	4066	2405	59.1	78.9	0.000	4361	1248	18.6	648.1	0.000
Middle (35–64)	5897	3674	62.3			4613	2303	49.9		
Old (65-)	460	369	80.2			327	266	81.3		
BMI										
<18.5	171	62	36.3	287.3	0.000	635	136	21.4	109.2	0.000
18.5–23.9	3528	1767	50.1			5519	1835	33.2		
24–27.9	4776	3106	65.0			2298	1275	55.5		
28-	1820	1431	78.6			676	477	70.6		
Abdominal obesity										
No	7379	4166	56.5	164.0	0.000	8268	3126	37.8	75.1	0.000
Yes	2919	2202	75.4			864	598	69.2		
Hyperuricemia										
No	8659	5263	60.8	24.4	0.000	8570	3424	40.0	53.1	0.000
Yes	1764	1185	67.2			731	393	53.8		

**Table 4:** Multiple logistic regression of variables associated with prehypertension

**Variables**	**Male**		**Female**	
	***OR***	**95% *CI***	***OR***	**95% *CI***
Age group				
Young (18–34)	1	—	1	—
Middle (35–64)	1.00	0.91–1.09	1.93	1.76–2.12
Old (65-)	2.71	2.12–3.48	7.29	5.40–9.83
BMI				
<18.5	0.55	0.40–0.76	0.68	0.55–0.83
18.5–23.9	1	—	1	—
24–27.9	1.72	1.57–1.89	2.02	1.82–2.25
28-	2.81	2.39–3.31	3.27	2.63–4.07
Abdominal obesity				
No	1	—	1	—
Yes	1.43	1.26–1.61	1.26	1.04–1.53
Hyperuricemia				
No	1	—	1	—
Yes	Not	significant	1.39	1.18–1.64

## Discussion

Despite evidences from a number of observational studies that demonstrate a relation between serum uric acid levels and hypertension, whether treating asymptomatic hyperuricemia contributes to the prevention of hypertension is still controversial. A prospective cohort study with a nested randomised controlled trial was designed to test the hypothesis that lowering uric acid levels by dietary intervention delivered by an educational package might prevent or postpone the occurrence of hypertension. Our study is intended as an exploration for the potential practical option of preventing hypertension besides providing proof of the physiological mechanisms involved.

In this prospective cohort study, four cohorts are being followed with the diagnosis of hypertension as the endpoint for each of the participant. The first question that will be answered is whether people with hyperuricemia are more likely to process from normal or high-normal blood pressure to hypertension or not. Recent experimental and clinical evidence supports the possibility elevated uric acid levels leading to hypertension [5,8,11-14]. In another prospective nested case control study, uric acid level was not associated with an increased risk of incidence of hypertension among older men and even in the subgroup of men who were < 60 years of age, this significant association was attenuated and no longer significant after further controlling for fasting insulin, triglycerides, and total cholesterol [11].

Additionally, whether lowering of serum uric acid confers protection against the development of hypertension remains untested. Regardless of the pathophysiological explanations, the observation that a high level of uric acid was associated with hypertension implies that it is possible to prevent or postpone the onset of hypertension by reducing serum uric acid. Randomised trial data on the effect of the intervention of lowering uric acid on the prevention or treatment of hypertension would be valuable. The nested RCT aims to identify the efficacy of a uric-acid lowering education package that is characterized as a purine-restriction lifestyle intervention. These include decreasing the consumption of protein, purines, and alcohol, as well as reducing obesity. Most uric acid comes from endogenous sources, therefore, diet is considered as playing a minor role in the treatment of hyperuricemia. It is still controversial whether asymptomatic hyperuricemia should be treated by lowering uric acid medications [15]. Although the uric-acid lowering effect appeared weaker than that of uric-acid lowering drugs, other associated health benefits would make lifestyle modifications important, particularly in the high risk of hypertension population [16,17]. Lifestyle modification intervention is considered as a practical strategy for the treatment of asymptomatic hyperuricemia. Healthy lifestyle interventions would form a basic therapeutic approach not only for hyperuricemia but also for hypertension,metabolic syndrome, and cardiovascular disease, though it is not easy to promote behavioural changes. It must be noted that a small reduction in the relative risk of hypertension can translate into large public health gains considering that more than 1.6 hundred million people in China suffer from hypertension. The outcomes of the RCT include controlling the occurrence rate of hyperuricemia and the transition rate to hypertension. In addition, a comparison of the transition rate to hypertension will be performed between the controlled hyperuricemia group and the uncontrolled hyperuricemia group.

Nesting an RCT within a cohort study is less likely to damage the overall observational nature of the study [18]. On the contrary, the RCT is potentially beneficial to the health of the participants and hence, it would increase the interest and enthusiasm of the staff and participants ensuring quality control during observations and data collection. It is possible that the RCT interventions would have altered the population cohort’s integrity, but for most interventions, the bias should be no different from that found in a normal observation study. Statistical analysis of the intervention will be conducted, where appropriate. A potential bias would be in the uric-acid lowering treatment for patients with symptoms of hyperuricemia. Subjects will be asked whether he or she had uric-acid lowering treatment in the last year, and if so this will be considered as a covariate during analysis.

There are several challenges to the implementation of this study. First, subject loss during the follow-up period is problematic in most cohort studies and it often leads to bias. A simulation study identified that seriously biased estimates of the odds ratios were obtained when observations were lost during the follow-up based on missing of random mechanisms [19]. The best way of dealing with loss during follow-up is to avoid it. Some efforts were made equally for each of group to avoid loss during follow-up. The employee whose employer had a long-term annual check-up contract with our centre was given priority for enrolling in this study with the expectation of the potential for a high compliance rate. At the start of this study, the names of two or more family members or friends who do not live with our participants were asked for enrolling in the study program. Small gifts were given as financial compensation for the time lost in completing the questionnaires for this study. Full-time personnel were hired for this task. Second, selection bias is built into the cohort studies. Participants who have hyperuricemia at the baseline probably differ in other important factors that are associated with the risk of hypertension. The information on other known risk factors of hypertension was collected for stratified and multivariate analyses. Over time, the exposure status (hyperuricemia) of the study participants can change. Serum uric acid will be measured annually to identify the potential change in its level. Third, non-adherence to highly effective recommendations can lead to intervention failure. Non-adherence rate is one of the outcomes that will be evaluated. Participants will be asked whether he/she has made a lifestyle change after the intervention and the potential factors associated with non-adherence will be identified. Factors associated with non-adherence in this study will be investigated according to the information filled in the questionnaire. Measures could be developed to ensure the implementation of intervention. In addition, a potential bias would be in the uric-acid lowering treatment for patients with symptoms of hyperuricemia. Subjects will be asked whether he or she had uric-acid lowering treatment in the last year, and if so it will be considered as a covariate during analysis. Finally, as this is a single-centre study, with less attention to details results may not be replicable in other settings. With the consideration of the beneficial intervention, one could apply the findings of this single-centre study only after comparing the context of the trial with his or her situation. The subjects in this study were employed by an employer who had a long-term contract with the Health Management Centre. One should be cautious when generalizing the research results to other populations.

Hyperuricemia was associated with prehypertension in subjects screened in our centre. Hyperuricemia was associated with prehypertension, and was independent of age, body mass index (BMI), and abdominal obesity in females; however, in males it was contrary. The relationship between gender difference and the association for uric acid with blood pressure was in line with the finding of a meta-analysis of 18 prospective studies on the association between hyperuricemia and hypertension [20]. Hormonal factors might influence uric acid level and they potentially have an impact on the relationships between uric acid and blood pressure [21-23]. Further investigation of selected medical conditions, uric acid, and hormonal factors in hypertension development is warranted.

Limitations of the current study include those inherent to any study of common lifestyle factors for both hyperuricemia and hypertension. In all dietary studies focusing on individual lifestyle factors, there is the potential for confounding due to the fact that many lifestyle factors are correlated. Information of potential confounding factors including sodium intake were collected as much as we can and multivariate statistical analysis will be adopted by considering multiple risk factors. Another potential limitation would be the size of the cohorts. As noted previously, there are many potential confounding factors should be taken into account to draw meaning conclusion. The current estimated sample size might attenuate the power of this study. The sample size would be re-calculated after preliminary results were obtained from the cohorts and a sequential design was considered.

Although there are a number of observational studies that report a on the relation between serum uric acid levels and hypertension, the impact of lowering uric acid on the prevention of hypertension is still inconclusive and requires further investigation. This RCT in the cohort study will provide important data on a group of subjects who are at a high risk of developing hypertension. A clear guideline regarding the choice of treatment of asymptomatic hyperuricemia is unavailable and this study aims to provide that.

## Competing interests

The authors declare that they have no conflict of interest.

## Authors’ contributions

GH and GW conceived the study and its design. KS and YW participated in the design of the study and drafted the manuscript. WL performed the statistical analyses and helped to draft the manuscript. QZ, HJ and WL participated in the acquisition of data. All authors critically revised the manuscript and read and approved the final manuscript.

## Authors’ information

Yuan Wang is Co-first authors.

## Pre-publication history

The pre-publication history for this paper can be accessed here:

http://www.biomedcentral.com/1471-2458/13/1069/prepub

## Supplementary Material

Additional file 1 Ethical approval of the ethical committee of the Tianjin Medical University. Click here for file

Additional file 2 Consent statement of participants. Click here for file
