# Supplementary material for: Does decreasing serum uric acid level prevent hypertension? – a nested RCT in cohort study: rationale, methods, and baseline characteristics of study cohort
Source: BMC Public Health. 2013 Nov 12;13:1069. doi: 10.1186/1471-2458-13-1069 (PMC3830560; doi:10.1186/1471-2458-13-1069)
Supplement: Additional file 1 — Ethical approval of the ethical committee of the Tianjin Medical University. [file 1471-2458-13-1069-S1.pdf]

天津醫科大學

人的医学伦理审查表

申请日期：2012 年 02 月 24 日　　天津医科大学伦理字 201235

项目名称：基于代谢组学方法尿酸在高血压前期进展为高血压过程中作用机制的研究		
项目负责人：芦文丽	职称：副教授	单位：公共卫生学院
项目联系人：芦文丽	电话：23542675	信箱：luwenli@tijmu.edu.cn
合作研究单位：		
研究者：李永乐 王媛 焦焕利 刘永哲 闫静 田庆伟 李文 张美琳		
请求审查类型：☑申请项目 ☐批准后项目 ☐延续项目 ☐委托项目		
研究项目来源：国家自然科学基金		

递交审查资料

☐实验方案　☑知情同意书　☐其他资料

包括：试验用品安全性资料，生产企业资质证明，试验用品提供者的资质证明。

涉及人的生物医学研究内容及研究方案摘要

本研究选择定期体检的高血压前期个体为研究对象（1500 人），在队列研究的基础上，选择高尿酸的高血压前期人群进行低嘌呤饮食指导为主的饮食干预，探索血尿酸水平在高血压前期进展为高血压过程中的作用及其可能的作用机制，寻找高血压预防和控制的新路径。研究所采取的干预为饮食干预，安全可靠。研究过程中，采取研究对象血样，采取过程与研究对象定期体检时间一致，采血量微小（5ml），不会对研究对象造成伤害，所采血样用于代谢组学测定。研究对象入组前将被详细告知研究目的，签署知情同意书。流行调查研究的内容不涉及研究对象的隐私问题。对所获取的研究对象个体信息，严格保密。

申报单位意见

主管领导签字：黄伟印国　　单位章：2012年 02月 24 日

伦理委员会审查意见

经审查"基于代谢组学方法尿酸在高血压前期进展为高血压过程中作用机制的研究"项目，将采取研究对象血液做干预效果评价，采取血液将在征得受试者知情同意后进行，经校伦理委员会审核，此项目符合卫生部《涉及人的生物医学研究伦理审查办法（试行）》及赫尔辛基宣言关于生物学人体试验的规定，同意开展研究

伦理委员会章： 2012 年 02 月 27 日

填表说明：

1、申请日期请填写拟交申请日期。
2、申请书中方格可在文字输入打印后，在选中的项目前用钢笔画√。
3、联系人为：本研究项目的联系人及电话。
4、请求审查类型中：延续审查课题为：一项课题需第二次审查的课题。
