# Supplementary material for: Does decreasing serum uric acid level prevent hypertension? – a nested RCT in cohort study: rationale, methods, and baseline characteristics of study cohort
Source: BMC Public Health. 2013 Nov 12;13:1069. doi: 10.1186/1471-2458-13-1069 (PMC3830560; doi:10.1186/1471-2458-13-1069)
Supplement: Additional file 2 — Consent statement of participants. [file 1471-2458-13-1069-S2.pdf]

《基于代谢组学方法尿酸在高血压前期进展为高血压过程中作用机制的研究》

参与者知情同意书

1、**研究背景和目的：**高尿酸血症是痛风的重要生化基础，此外它还很可能与动脉粥样硬化，冠心病、高血压、血脂异常及胰岛素抵抗等有密切关系。随着经济的发展，人们的饮食习惯、生活方式发生了巨大改变，代谢综合征、痛风患病率快速增长，且与心血管病的发病联系亦日渐明确，目前已成为公认的世界性公共卫生问题。本研究主要探讨通过饮食干预降低尿酸水平对于预防高血压病发生的理论意义和实用价值。

2、**研究方案简介：**本研究以患高尿酸血症，并处于高血压前期的个体研究对象，探索血尿酸水平在高血压前期进展为高血压过程中的作用，考证血清尿酸水平与高血压发生的时间顺序问题，分析尿酸是否为高血压病的独立危险因素及其可能的作用机制，寻找高血压预防和控制的新路径。

3、**参与者的责任和义务：**您在参加体检的的时候要协助完成问卷调查，问卷的问题包括一些与健康和生活习惯相关的问题，如吸烟、饮酒、体力活动等，尤其是需要认真填写 7 天的膳食摄入记录，使我们能够更准确的评估您的膳食习惯，真实的反映出本实验需要了解的真相。我们会向您提供降低血尿酸水平的饮食调整建议，为了您的健康，请您根据该建议积极调整自己的饮食习惯。研究过程中，我们会尽量根据您每年体检的时间安排您的调查和访谈，在第一次体检后六个月左右，我们将派专业人员到您家中再次进行一次补充采血取样和补充问卷调查，具体时间我们将通过电话与您协商，恳请您一定要留下确切的地址和联系方式，并配合我们做好工作。

4、**参与者的纳入和排除标准：**参与者为年龄在 40-60 岁之间的高血压前期（未服用抗高血压药物情况下，血压水平处于 120-139/80-89mmHg）的体检对象。排除合并临床症状者，即脑血管病：缺血性脑卒中史，脑出血史，短暂性脑缺血发作史；心脏疾病：心肌梗死史，心绞痛，冠状动脉血运重建，充血性心力衰竭；肾脏疾病；糖尿病肾病；肾功能衰竭；恶性肿瘤患者。

5、**参与者的获益：**本研究所有参与者，在认真填写调查表后，不仅拿到了自己的体检结果，而且还会得到一份我们精心给您准备的小礼物和饮食习惯评估建议。而研究最终的结论，会为健康的长久、家庭的幸福做出贡献。

6、**参与者的风险：**本研究不对任何参与者造成任何危险。包括实验所需的血、尿液标本，也只是在正常体检的血、尿液标本上增加了微小的量。采血过程中可能有皮肤敏感或感染现象，但发生概率非常低。

7、**保密措施：**本项研究的记录将严格保密，不会公开泄露参与者的姓名。本研究的结果形成的论文在公开出版的期刊上发表,研究单位可以进行成果鉴定。

在研究过程中，如果有任何疑问或不理解的事情，参与者可向负责研究的代理人要求解释任何需要解释的情况。

参与者声明：

我已与本项试验的研究者详细讨论并了解过本项研究的目的、过程、方法、风险和受益、保密性.并仔细阅读以上有关说明后，经过充分时间的考虑，我自愿成为此项研究的参与者，同研究者全面合作，积极配合研究人员进行本项研究。

参与者签名：　　　　　　　　　　联系电话：　　　　　　　　　　日期：

研究者签名：　　　　　　　　　　联系电话 ：　　　　　　　　　　日期：
